# Supplementary material for: Unraveling the Guardians of Growth: A Comprehensive Analysis of the Aux/IAA and ARF Gene Families in Populus simonii
Source: Plants (Basel). 2023 Oct 13;12(20):3566. doi: 10.3390/plants12203566 (PMC10610179; doi:10.3390/plants12203566)
Supplement: Supplementary file 1 [file plants-12-03566-s001.zip › Supplemental Figures.pdf]

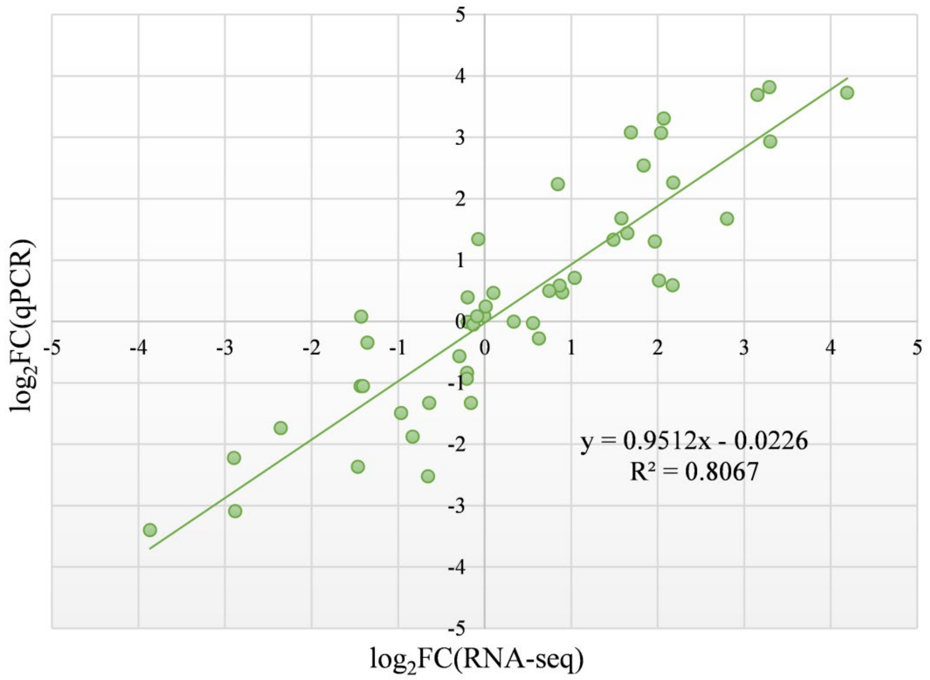

Supplemental Figure S1. Correlation between relative expression of qPCR data and FPKM values of RNA-seq data. X-axis represents $\log_2$FC(qPCR), Y-axis represents $\log_2$FC(RNA-seq).
